# Experiences of lifestyle changes among Thai older adults six months after applying the Plan-Do-Study-Act (PDSA) cycle

**DOI:** 10.1186/s12877-024-05481-5

**Published:** 2024-10-31

**Authors:** Manothai Wongsala, Sirpa Rosendahl, Els-Marie Anbäcken, Pornpun Manasatchakun, Jessica Holmgren

**Affiliations:** 1 Boromarajonani College of Nursing Nakhonratchasima, Nakhon Ratchasima, Thailand; 2 School of Health, Care and Social Welfare, Mälardalen University, Västerås, Sweden; 3 School of Health Sciences, University of Skövde, Skövde, Sweden; 4 School of Health, Care and Social Welfare, Linköping University, Mälardalen University, Västerås, Sweden; 5 Boromarajonani College of Nursing Chiang Mai, Chiang Mai, Thailand

**Keywords:** Active ageing, Lifestyle change, PDSA cycle, Thai older adult, Qualitative method

## Abstract

**Background:**

Thai older adults are valuable resources in their society. The Thai health service system is challenged when it comes to ensuring that older Thai adults can continue to live healthy and independent lives in society. It is of great value to support independence and improve older people’s active ageing. Promoting lifestyle changes by applying the Plan-Do-Study-Act cycle (PDSA cycle), at group meetings in a municipality context, is a way of focusing on active ageing. This study aims to describe older adults´ experiences of lifestyle change six months after finishing group meetings applying the PDSA cycle.

**Methods:**

A qualitative approach with individual interviews and a qualitative content analysis were used with 12 Thai older adults who participated in the meetings applying the PDSA cycle.

**Results:**

Six months after finishing applying the PDSA cycle, some older adults kept their individual goals and were influenced by their family surroundings. They also formulated additional goals. Three categories and six sub-categories emerged: Keeping individual goals, influenced by the surroundings, and formulation of additional goals were the overall categories.

**Conclusions:**

These Thai older adults showed that they had the ability to make lifestyle changes with the support of the PDSA cycle, but not all maintained their planned activities after six months. The question is how healthcare professionals and the surroundings, may further support and motivate these people to maintain these changes based on their own preferences in a sustainable way.

## Background

The older Thai population is a valued resource both in their families and community. The health and welfare system aims to ensure that older adults maintain their good living status and have the potential to continue contributing to society [1]. Maintaining a good living status among older adults is an important mission of the health and welfare system in Thailand. The Thai population aged 60 years and older was 19.57% in 2021 and is increasing [2]. The Thai government has agreed that active ageing, as a concept and as a practice should be part of the national agenda [3]. Active ageing and its principles have been presented by the World Health Organization to maintain and enhance these aspects as people age. This is through three basic pillars consisting of health, participation, and security [4]. These pillars of active ageing have been used in policy and practical levels globally [5]. However, adjustments are required to suit different social contexts [6].

A Swedish study indicated that the activities provided to older adults were not as important as the socialization that the activities entailed. It is possible that participating in various social interactions contributes to maintaining and enhancing active ageing. Older adults leading meaningful activities themselves, has also been suggested [7]. Chi and colleagues [8] conducted an activity programme consisting of lectures about ageing and health, physical activities, and social activities. They found that the programme could improve mental health, social participation, and active ageing among Taiwanese older adults. They also pointed out that group activities at a community centre had more benefits than individually based activities. A study conducted in Spain-France- Andorra by Blancafort Alias and colleagues [9] mentioned that group activities were commonly used for a wide range of purposes. They contributed to better outcomes regarding lifestyle change and social support. However, all activities were led by the community centre staff and did not include encouraging older adults to maintain and enhance lifestyle changes themselves. Most activities to promote active ageing for Thai older adults, are provided at a group level in healthcare units. There are for example 29,359 senior citizen clubs and 2,303 senior citizen schools [10]. These may take the form of physical training, recreation activities related to religion and lecturing about health [11]. Previous research shows that activities provided for older adults in Thailand and Taiwan are often initiated and suggested by healthcare professionals.

Active ageing could be maintained and enhanced by changing lifestyle [12]. However, it may be a challenge to promote and motivate an individual to think about change and really act. Raihan and Cogburn [13] highlighted the stages of change in their research. Changes begin with thoughts that something can be improved. However, persons may consider that their current lifestyles are good enough. A change is therefore difficult to initiate. Moreover, a change may also be complicated to maintain. Previous studies show that ageing persons tend to select an activity, and thus reduce the number of activities they participate in [14, 15]. Therefore, tools and activities that support and encourage older adults’ potential capacity, to manage lifestyle changes by themselves are desirable. One such tool which is used in this study is the Plan-Do-Study-Act cycle (the PDSA cycle). The P stands for Plan, which may encourage older adults to think independently about what needs to be changed, to identify a goal leading to a lifestyle change, and plan how to carry it out. The D stands for Do, which is the active step of carrying out an activity to reach a goal. The S stands for Study, which provides a chance to evaluate if the change is an improvement or if the activity of change needs to be adjusted to be an improvement. The A stands for Act, which represents adaptations, and refinements of the development process [16]. This tool may provide both small-scale changes and more extensive changes, and it supports the possibility of achievement in the pursuit of a change. In addition, the cycle also minimizes the risk of returning to old behaviours [17].

The PDSA cycle tool was originally generated for industry to improve productivity but has also been applied and used in healthcare organizations [16, 18]. A study conducted in the United States found that this tool could be applied to improve the quality of healthcare systems [19]. Another study in Canada showed that the PDSA cycle had potential as an intervention for improving quality in specific areas such as reducing unnecessary urinary catheters in hospital [17].

PDSA has been shown to be a tool with the potential to support improvement in both organizations and individuals. Individual level improvements are particularly evident since PDSA can support people in a systematic and process-like way. Even with a smaller goal in everyday life, PDSA gives a person a clearer direction to reach a goal. It has been reported that the PDSA cycle, has been used for lifestyle changes among individual older adults, in a group context in Sweden [20], England [21], and in Thailand [22]. However, studies on applying the PDSA cycle for individual lifestyle changes to promote active ageing are limited. The studies reported individual lifestyle changes at a moment in time and lacked knowledge about whether the changes remained in place over time. Although the PDSA cycle may support lifestyle changes, changes often take a long time to become part of life [23]. In addition, lifestyle changes in relation to this tool could be influenced by many factors over time. Therefore, it is of particular interest to explore older adults’ experiences of applying the PDSA cycle to promote active ageing six months after finishing LS-meetings. Based on the background presentation, the focus will be on a Thai context.

## Methods

This study is part of a larger project. In a previous study, the PDSA cycle was used to support older adults to manage changing their own lifestyles. This current study aims to describe older adults´ experiences of lifestyle changes six months after finishing group meetings applying the PDSA cycle. A qualitative follow up approach was used to obtain an understanding of the studied phenomenon by exploring peoples’ experiences. Data were collected by individual interviews, and analysed by qualitative content analysis as described by Graneheim and Lundman [24].

### Setting

The study was conducted in a province in northeast Thailand, which is a part of the so-called Isan culture. This area represents general Thai culture, and it has a substantial proportion of older adults [25]. The Isan region includes a sub district health promotion hospital, a senior club, and a school for older adults. These units usually provide activities at a group level to maintain and enhance the health of older adults in the area. This unit represents both urban and rural lifestyles, since it is situated in a sub-urban area of a large city. The Isan region sub district health promotion hospital, promotes public health for the population in three surrounding villages, and organizes group meetings to some older persons. This study was part of a larger research project that aimed to encourage lifestyle changes with the support of the PDSA cycle. The group meetings were called Lomwong Saangsook meetings (LS-meetings). Lomwong Saangsook means “to collaborate to build happiness” in Thai [22].

### Participants

Participants were Thai older adults aged 60 or above, without severe illness, able to communicate without difficulties. They were the same participants as the previous study [22] and six months before had participated in LS-meetings using the PDSA cycle. They were invited to participate in the LS-meetings by healthcare professionals working at the Isan region sub district health promotion hospital. In this study, their experiences were followed up at an individual level. From the beginning, a total of 15 participants attended the LS-meetings. However, three of them dropped out. Two communicated that they were too busy to participate in the interviews and the third participant moved away. Therefore, 12 participants were included in the current study (see Table 1).

**Table 1:** Participant characteristics

Characteristics	Number
Age	
60–65	5
66–70	3
71 and above	4
Occupation	
Gardener	2
Self-employed	2
Retired	6
Housekeeper	2
Gender	
Male	6
Female	6
Education	
Primary school	11
Secondary school	1
Married status	
Married	6
Widowed	6

The participants lived in three villages inside the healthcare unit catchment area. None of them had a severe illness or communication limitations. The participants were informed about the overall purpose and data collection methods employed in this study as well as the estimated time required for participation. Information was given both orally and in written form. The informants were informed that they had the right to withdraw from the study without explanation at any time without negative consequences. The participants were asked to sign a consent form before they were interviewed.

### Data collection

The individual interviews, focusing on participants’ experiences of lifestyle change six months after the last LS-meetings were conducted. Although the already completed LS-meetings were group activities that focused on individual goals, most activities were conducted individually. This created the conditions for conducting individual interviews. The participants selected the place and decided the interview time. The interviews were conducted in Thai by the first author and were audio-recorded. Questions were asked during the interviews to encourage the participants to express their experiences independently. The questions were designed to provide a structure, to ensure focus on the research aim and to obtain as much information as possible. Each interview covered 30 to 50 min, and the average interview lasted 35 min. The time included greetings and some warmup talks. The main interview questions were: *Could you please talk about your participation in the LS-meetings? How have you carried out your personal plans to reach a specific goal? Could you please describe your experiences when following your plans? How have you achieved your goals to promote health*,* security*,* or participation in your daily life? What kind of changes occurred when you followed your plan? Did you experience any kind of challenges*,* and if that was the case*,* could you please share something about these issues? How have you achieved your goals to promote health*,* security*,* or participation in your daily life? Could you please give me some concrete examples*,* related to these aspects?* During the interviews, the participants were treated with respect as seniors, according to the traditional Thai way.

### Data analysis

The data were analysed using qualitative content analysis according to Graneheim and Lundman [24]. The interviews were transcribed verbatim by the first author since they were in Thai. Three interviews were then translated into English, so that the English-speaking authors could check the accuracy of the content. One of the authors who spoke advanced Thai and English, double checked the translated transcripts. If there was any uncertainty about the understanding of the content, then this was checked and discussed between the authors, until a shared understating was reached. Data analysis was then conducted (see Table 2).

**Table 2:** The data analysis process

Steps of analysis [24]	Analysis of the individual interviews
Obtain a sense of the whole	The interview transcripts were read in an overall manner, to obtain a sense of the whole.
Identify meaning units	Meaning units that corresponded to the aim in this study, were identified from a sentence or several sentences.
Find out the core of the meaning units	Meaning units were condensed by shortening the identified texts, to keep the core meaning in relation to the aim.
Label meaning units with codes	The condensed meaning units were labelled by short words, in the form of codes.
Abstract codes into sub-categories, based on similarities and differences	The codes were sorted into subcategories based on their similarities and differences.
Abstract sub-categories with similar content to main categories	Sub-categories with similar content were abstracted into main categories.
Check and revise categories and sub-categories	The consistency within and between sub-categories and categories were discussed among the authors. Some adjustments were made until an agreement was reached about the final structure of the main categories and sub-categories. This procedure was preceded by sub-categories and categories being discussed by the authors in both Thai and English separately.

### Ethical considerations

This study was approved by the ethical review boards in Sweden and Thailand. The research process in this study takes into account, the guidelines of the WMA Declaration of Helsinki - Ethical Principles for Medical Research Involving Human Subjects [26]. Fictive names have been used in the findings section to protect study participants. All data were kept in a password protected computer at a college in Thailand during the analysis processes. The data will continue to be stored in the first author’s college, according to current local guidelines.

## Results

The findings consist of three main categories and six sub-categories as shown in Table 3. Thai older adults shared their experiences of lifestyle change on how they had *kept their individual goals*,* being influenced by the surroundings*, and how they had *formulated additional goals* by themselves.

**Table 3:** Main categories and subcategories of the findings

Main categories	Sub-categories
Keeping individual goals	Attention to the plans
	Adjusting the way to suit daily living
Influenced by the surroundings	Facing the obstacles
	Receiving support from others
Formulation of additional goals	Getting inspiration from others
	Initiating new goals based on new knowledge or insight

### Keeping individual goals

During the LS-meeting time, each participant had set their own individual goals and decided how to carry them out. The first main category described how the participants kept their individual goals and how they carried them out.

### Attention to the plans

After finishing the LS-meeting, the participants continuously carried out their individual goals, but for some the frequency of carrying out the plan gradually decreased over time. However, they described that they had carried out activities more often than they had done before they participated in the LS-group meetings.

*I have been following the diet plan. But it is hard to do so. I knew what I was supposed to do*,* but sometimes I could not find multiple dishes. I tried to circularly rotate the dishes from chicken to pork*,* to beef*,* and to fish. I also tried to consume vegetables every day (Lei*,* 77)*.

*Sometimes*,* I followed the plan. Mostly*,* I did only when I wanted to or when I recalled it*,* but I think I did it more often (Po*,* 70).*

The participants were reminded of experiences from participation in LS-meetings. They paid attention to their own goal when they carried out daily activities. When most participants carried out daily activities they were reminded of experiences from participation in the LS-meetings. Their own speech to the meeting members, and knowledge they had gained at the LS-meetings reminded them to continue planned activities.

*Sometimes I drank black coffee*,* but it did not have a good taste. I know it is good for health to reduce sugar and cream. When I remembered the meetings*,* I added less sugar and cream to coffee (Saw).*

*I now realize how much I should drink a day and if it is enough. I do it because I think it is good for myself*,* but I just sometimes think about the meetings when I do something relating to what I said in the meetings (Tim*,* 70)*.

Although the participants were encouraged to make lifestyle changes, some of them decided to continue the same activities they had done as daily routines. However, experiences from participation in the LS-meetings reminded them to carry out daily activities in a different aspect. Some participants appeared to think about how the activities benefitted them, although they did not change daily activities.

*I have done the same as my routine*,* but I think about the reasons I do it. I also think about how to do it better. I recognized what the moderator said at the meeting about why it is good for me (Sam*,* 72).*

Some of the goals defined by the older adults themselves were not new activities, especially the goals connected to the participation pillar of active ageing. They considered that continuing the same activities were fine and still benefited them. Most of the male participants confirmed they would continue supporting the temple while women confirmed they would participate in the community events as they used to. They described that they had done this before participating in the LS-group meetings.

### Adjusting the way to suit daily living

As time passed, the individual goals which were defined in the LS-meetings were still thought of as being significantly important. However, goals needed to be adjusted to suit daily living. Participants had strong intentions and tried to carry out the activities expressed at the LS-meetings. However, if the new activities were not part of their regular lifestyle, they experienced difficulties and the methods used to carry out the plans were changed.

*I intended to save money a little bit every day. But my priority is to give my granddaughter lunch money to school. It is better to save 200 baht from the Elderly Pension Welfare Programme instead (Lei*,* 77).*

The participants tried to keep their own individual goals, although the ways they carried out them might change to suit their regular daily living. They were also reminded by their own sayings and mini lecture by the LS-meeting moderator. They gained knowledge that made them concerned about the initiated individual goals and benefits of each daily activity.

### Influenced by the surroundings

During the time for doing lifestyle changes the older adults found both obstacles and support. This second category shows that the obstacles could affect the way plans were carried out. Some decided to stop carrying out the plan while others decided to compromise by adjusting it.

### Facing the obstacles

Maintaining daily routines while living in extended families was sometimes difficult. A woman described that she could not carry out her individual plan continuously when family members influenced her daily living.

*I intended to reduce Monosodium glutamate (a flavour enhancer) in our meals. My children said that it was OK to avoid it*,* but they did not like it. We finally found a happy middle way. Since then*,* I have added around 50% less (Kan*,* 65).*

When facing some obstacles, some Thai older adults in this study adjusted the way to achieve the plan while others changed the way completely. One woman talked about her intention to exercise using aerobic dance. However, the music annoyed her ill husband, and she therefore terminated this form of exercise. Older adults might also have internal obstacles such as time and physical limitations.

*I would like to do more arm swings*,* but I have back pain. I tried to do it a few times in many sets instead of so many times continuously. I will try*,* but I will stop if I feel back pain (Mac*,* 63).*

Most obstacles found during carrying out the plans related to the people surrounding them, especially family members. Older adults made efforts to deal with these obstacles by adjusting to surrounding people but found that a better way was to adjust themselves.

### Receiving support from others

Some participants were supported by their family to make lifestyle changes. Family members provided materials, gave up their free time, and sometimes asked how activities had gone. This was a physical as well as mental support.

*My grandchild who gave me an indoor bicycle always asked me how I had exercised with it (Kan*,* 65).*

The participants mentioned supportive factors that encouraged them to continue lifestyle changes. One participant said that nice comments by the family supported participation in LS-meetings and encouraged continued implementation of the plan.

*My daughter thanks me for what I do to take care of myself. She said that I look healthier and fresher. It felt good that she was supporting me. If she did not agree with me about attending the meetings*,* I would feel uncomfortable (Tim*,* 70).*

Since most of them lived in extended families, family members could influence their ways of carrying out the plans. Therefore, family is one important supportive factor while carrying out the plan at home.

### Formulation of additional goals

In the last category, it emerged that once the LS-meetings were finished, the participants continued to carry out the plans. However, as time passed some activities were not followed through or adjusted in a way that meant they could be performed, and new goals were also formed. The participants described that they would like to keep carrying out their own plans which were defined at the beginning. However, when they found that the activities were not suitable in their living context, they accepted changing, adjusting, or finishing the activities. They also experienced that it was beneficial to copy other activities and form a new lifestyle according to the knowledge they learned from the LS-meetings.

### Getting inspiration from others

The participants sometimes copied other members’ activities and goals. They did not mind if the activities followed their own plan or not. The beneficial activities defined by others were a source of knowledge in setting additional personal plans.

*I stretch my legs like this and try to touch my feet (show how to do). I think it is the same as Mr. Sam did. I stretch and twist like this. I have done this after waking up every day (Jet*,* 64).*

Another participant said they did not drink enough water. Drinking enough water was not part of their plan at the beginning, but they said it was fine to set it as a new goal. The informant also explained they had never measured how much water they had drunk in everyday living.

*I fill bottles with water to drink*,* so I know how much I drink a day. Before the meeting*,* I just drank when thirsty and never thought about how much I drank (Lei*,* 77).*

When the participant was asked if this activity was their individual plan from the beginning, they said that it was fine to be inspired by others since the activity had health benefits.

*I think it is fine to copy ideas from others. They are not protected by copyright*,* are they? (laughs). All humans want to be healthy*,* but we might just live and do nothing. When we have things like those (the meetings)*,* we gain knowledge and learn what we should do to be healthy (Lei*,* 77).*

The participants mentioned that they also copied other activities which were expressed at the LS-meetings. When asked to set the goal during applying the Plan step of the PDSA cycle, they sometimes had difficulty formulating goals. Therefore, some of the informants sometimes copied other members. This strategy also continued after finishing the LS-meetings.

### Initiating new goals based on new knowledge or insight

Most participants mentioned that they had received new knowledge during the LS-meetings. They said that they thought about what the moderator had advised them to do for a better lifestyle. They had gained knowledge to take care of themselves as an older person. Thanks to new knowledge, they initiated new goals, formed a plan, and set their own course to achieve these goals themselves. The participants continued carrying out some of their own plans, they copied the activities from others, and followed advise or examples given by the moderator. They selected and carried out activities that benefitted their daily living.

*At the meetings*,* we had good suggestions about how to take care of ourselves by the moderator. We received good knowledge that benefits our health. I have done so*,* following that (Sam*,* 72).*

The participants could initiate a new goal and create new activities that differed from their own individual goals, formulated at the beginning. They also described that they were concerned sometimes about whether the activities were good or bad for their health. However, the knowledge they gained from the mini lectures provided by the moderator, and insight on how to improve their own lifestyle mostly inspired them to form new individual goals.

## Discussion

This study described older adults´ experiences of lifestyle changes six months after finishing the LS-meetings and applying the PDSA cycle. According to the stage of changes [13], as older adults found reasons to change their lifestyle, the process of change was started.

Reaching a specific goal was achieved by having the ability to adjust individual daily living, to follow the steps of the PDSA cycle automatically. This indicates that Thai older adults may have had the insight to make these changes themselves. Findings reveal that the final goals of applying the PDSA cycle are not fixed but adjusted and tailormade by the older persons to reach their individual goals which were planned at the previous LS-meetings. The success factor seems to be the capacity of participants to plan, carry out, evaluate/learn, and then decide how to continue activities that may enhance active ageing. The findings also show that older adults selected the best satisfying activities to motivate themselves to change their lifestyles. This is in accordance with what Komp and Aartsen [14] raise in their research. When Chi and colleagues [8] reported that programs provided by health staff could improve active aging, this current study presents a difference. Current findings are in line with a previous study [7] which suggested that leading meaningful activities by older adults themselves caused better outcomes.

Application of the PDSA cycle was influenced by social surroundings. Both supportive factors and obstacles were found. The most obvious of these was the influence of the family since most of the participants lived in extended families. In contrast, the studies by Gilbertson and Batty [21] and Nilsson [20], do not mention any kind of support from families between group meetings. Dealing with obstacles, adjusting the way of carrying out the plans, and defining new goals could be seen as older adults increasing their potential to manage lifestyle changes to enhance active ageing by themselves. Although the LS- meetings were finished, older adults could decide to enhance active ageing independently. Creating new goals could be seen as older adults starting new PDSA cycles by themselves in accordance with Taylor and colleague [16]. Those participants lacking support from their families, had the support of peers in the LS-meetings who could make a substantial difference in helping these participants to succeed with their PDSA application.

Apart from learning from the group members, the participants also received new knowledge from the moderator in the LS-meetings. The Thai older adults trusted and followed the health care professionals. Positive results were shown by the participants when they said that they were encouraged by the moderator´s words during the LS-meetings. When comparing activities provided to promote active ageing in Thailand [11], applying the PDSA cycle may promote older adults’ capacity to manage lifestyle change by themselves. Considering the older persons themselves chose the activities and followed them up, this may create the conditions for sustainable active ageing.

Considering lifestyle change according to the basic pillars of active ageing [4], the health pillar was mentioned the most, compared to the other two pillars, participation, and security. During applying the PDSA cycle, the health pillar was offered to the previous conducted LS-meetings as the first topic. Appropriate diet and exercise which are a part of the health pillar, were also the earliest topics offered and received much focus in the LS-meetings compared to the other two pillars [22]. Thai older adults often consider that the health aspect is the most important for them [27].

The pillar participation seemed to be a bigger challenge to consider, in terms of lifestyle change. Furthermore, the participation pillar might need the involvement of other people while health could be a personal activity. As for the pillar security, it was a challenge to think about changing lifestyle for security since older adults expect that this is something that should be provided by others, such as the welfare system present in Thai society.

Another factor is that the interviewer may have been perceived as being more of a healthcare provider than a researcher. Therefore, health issues may have been more natural to talk about. It may be the case that the pillars of participation and security, were less evident since the study focused on experiences of lifestyle changes, which may be strongly associated with the health pillar.

As in all studies, this study has both strengths and weaknesses. The strength of this study is being a part of a whole project which is a pioneering study of applying the PDSA cycle to promote health behaviour in older adults in Thailand. An additional strength is that the implementation of the PDSA cycle in ageing and health contexts, provides some kind of structure for making lifestyle changes. That could compensate the limitations associated with traditional implementation to promote active aging in Thailand. Since the interviewing was followed up six months after the LS-meeting, some participants dropped out. However, there are still twelve participants that have been interviewed. Following up could be a strength of this study since it explored how older adults maintain their new lifestyles.

### Methodological considerations

Older Thai adults have been able to share their experiences in a qualitative way, and the aim has been answered. It has been a bit of a challenge to create and follow the interview questions. The questions may have been perceived as partly leading and too comprehensive, related to the fact that it is an art to be able to conduct qualitative interviews. The questions also included the active ageing pillars, which may have been difficult to answer since some of them may appear as far-fetched. The fact that the first author has a background as a healthcare professional, may have had an impact on the data collection in both a positive and a negative way. It may be that the informants had ideas about him, which caused them to respond in a way that they would not have done to a person without this background. On the other hand, they may also have felt trust and confidence which meant that they shared their experiences in an open way. The author who collected the data tried to assume the role of a researcher and thus curbed his role as clinician. He did this by, among other things, listening extra carefully, and asking follow-up questions even if he had a pre-understanding of the studied phenomenon. Moreover, some of the data were translated from Thai to English which strengthened the credibility of the data analysis and findings. Since the interviewer is the one who holds group meetings, participants may express positive opinions to maintain a good relationship. The interviewer reduced this bias by carefully showing the role of the interviewer as a researcher and the participants can be freely given and will not affect the service received. The interviewers let participants talk freely.

## Conclusions

This study shows that Thai older adults have the capacity to keep their individual goals which were defined in some previous LS-meetings when applying the PDSA cycle. The ways to reach the goals were adjusted, to be suited to daily living in a flexible way. They faced influences from their surroundings, and these could form both obstacles or supportive factors. The knowledge older adults gained during the LS-meetings inspired them to define additional new goals by themselves. The LS-meetings applying the PDSA cycle could promote capacity to manage lifestyle change by older adults themselves. The application of the PDSA tool proved to be suitable to be applied on both a group and individual level in this study. This is relevant in the Thai context, since promotion regarding active ageing in Thailand is often provided in groups in communities. The contributions of this study are having knowledge that Thai older adults have the capacity to manage their lifestyle changes by applying the PDSA cycle when supported by their surroundings. This knowledge should be considered when providing activities in health promotion based on initiatives by older adults themselves. The knowledge was expected to inform Thai healthcare providers who promote active ageing based on encouraging self-management especially when applying the PDSA cycle to promote health behaviours in older adults. However, since there seems to be a paucity of studies of PDSA cycle application regarding active ageing, future research in other settings is needed. In addition, how the PDSA cycle promotes active ageing for older adults with health conditions such as diabetes or hypertensions is also needed.

## Data Availability

Data will be stored in the college of the first author, according to local guidelines. The datasets used and/or analysed during the current study available from the corresponding author on reasonable request.

## Abbreviations

PDSA cycle: Plan-Do-Study-Act cycle
LS: Meetings-Lomwong Saangsook group meetings
